# A novel transposon construct expressing PhoA with potential for studying protein expression and translocation in *Mycoplasma gallisepticum *

**DOI:** 10.1186/1471-2180-12-138

**Published:** 2012-07-08

**Authors:** Indu S Panicker, Anna Kanci, Chien-Ju Chiu, Paul D Veith, Michelle D Glew, Glenn F Browning, Philip F Markham

**Affiliations:** 1 Asia-Pacific Centre for Animal Health, Faculty of Veterinary Science, The University of Melbourne, Parkville, VIC, 3010, Australia; 2 Melbourne Dental School and Bio21 Institute, The University of Melbourne, Parkville, VIC, 3010, Australia

**Keywords:** *Mycoplasma gallisepticum *, Lipoprotein, Membrane protein, Reporter gene, *phoA *, Alkaline phosphatase

## Abstract

**Background:**

*Mycoplasma gallisepticum * is a major poultry pathogen and causes severe economic loss to the poultry industry. In mycoplasmas lipoproteins are abundant on the membrane surface and play a critical role in interactions with the host, but tools for exploring their molecular biology are limited.

**Results:**

In this study we examined whether the alkaline phosphatase gene (*phoA *) from *Escherichia coli * could be used as a reporter in mycoplasmas. The promoter region from the gene for elongation factor Tu (*ltuf) * and the signal and acylation sequences from the *vlhA *1.1 gene, both from *Mycoplasma gallisepticum *, together with the coding region of *phoA *, were assembled in the transposon-containing plasmid pISM2062.2 (pTAP) to enable expression of alkaline phosphatase (AP) as a recombinant lipoprotein. The transposon was used to transform *M. gallisepticum * strain S6. As a control, a plasmid containing a similar construct, but lacking the signal and acylation sequences, was also produced (pTP) and also introduced into *M. gallisepticum *. Using a colorimetric substrate for detection of alkaline phosphatase activity, it was possible to detect transformed *M. gallisepticum *. The level of transcription of *phoA * in organisms transformed with pTP was lower than in those transformed with pTAP, and alkaline phosphatase was not detected by immunoblotting or enzymatic assays in pTP transformants, eventhough alkaline phosphatase expression could be readily detected by both assays in pTAP transformants. Alkaline phosphatase was shown to be located in the hydrophobic fraction of transformed mycoplasmas following Triton X-114 partitioning and in the membrane fraction after differential fractionation. Trypsin proteolysis confirmed its surface exposure. The inclusion of the VlhA lipoprotein signal sequence in pTAP enabled translocation of PhoA and acylation of the amino terminal cysteine moiety, as confirmed by the effect of treatment with globomycin and radiolabelling studies with [^14^ C]palmitate. PhoA could be identified by mass-spectrometry after separation by two-dimensional electrophoresis.

**Conclusion:**

This is the first study to express PhoA as a lipoprotein in mycoplasmas. The pTAP plasmid will facilitate investigations of lipoproteins and protein translocation across the cell membrane in mycoplasmas, and the ease of detection of these transformants makes this vector system suitable for the simultaneous screening and detection of cloned genes expressed as membrane proteins in mycoplasmas.

## Background

Mycoplasmas are prokaryotes in the class *Mollicutes * and are characterised by the absence of a cell wall [1]. Mycoplasmas cause disease in a number of animal species and are able to survive and persist in the face of host defences, even though they possess a relatively small genome and are bounded by a single protective plasma membrane. The recent chemical synthesis and cloning of whole mycoplasma genomes has also drawn attention to the possibility of creating synthetic cells and genetic manipulation of the smallest bacterial genomes [2,3]. The proteins within the single limiting membrane of mycoplasmas fulfill many of the critical functions related to morphology, nutrient transport, environmental adaptation and colonisation of the host [4].

Many of the surface proteins of mycoplasmas are amphiphilic and/or lipid modified and some have been shown to be components of solute transport systems or involved in antigenic variation and adherence, while the functions of many others remain unknown [5-7]. Mycoplasmas possess an unusually large number of lipoproteins, which are anchored to the cell membrane by a lipid moiety, with the polypeptide moiety exposed on the cell’s outer surface [8]. Lipoprotein signal peptides are cleaved by signal peptidase II at a conserved motif preceding the amino terminal cysteine of the mature lipoprotein. The significance of mycoplasma lipoproteins in interactions with the host emphasises the need to better understand how they are processed, and the mechanisms controlling their expression [4].

*Mycoplasma gallisepticum * is a major poultry pathogen, causing chronic respiratory disease in chickens, infectious sinusitis in turkeys and conjunctivitis in house finches [9,10]. It has a worldwide distribution and causes severe economic losses in the poultry industry. Vaccination of the flock is a necessity to control mycoplasmosis in commercial poultry farms. The live vaccines in use at present are F strain, 6/85 and ts-11 [11]. Although effective and widely used at present, these vaccines could be modified to act as vaccine vectors to deliver other antigens and thus be the basis of multivalent vaccines.

Although the genome of *M. gallisepticum * strain R_low_ has been sequenced [12], the lack of genetic systems for mycoplasmas in general impedes our ability to study their molecular biology. The use of UGA as a tryptophan codon in mycoplasmas also makes it tedious to use heterologous hosts such as *Escherichia coli * for expression and characterisation of cloned mycoplasma sequences [13]. Molecular tools such as reporter gene systems suitable for studying lipoprotein processing and expression in mycoplasmas are necessary.

The *E. coli * ß-galactosidase gene (*lac *Z) has been used to identify gene promoters and detect genetic regulatory elements in *M. gallisepticum * and *Acholeplasma * species [14,15], and as a reporter gene in combination with the *vlhA * 1.1 promoter in *M. gallisepticum * S6 [16]. A major drawback of the use of ß-galactosidase (ß-Gal) as a reporter is its limited ability to pass through the bacterial cytoplasmic membrane [17]. When the gene for an exported protein is fused to *lacZ *, the hybrid protein is membrane bound and such proteins have very low ß-galactosidase activity [18]*.* Green fluorescent protein (GFP) has been used to identify promoter sequences in DNA libraries of *Mycoplasma pneumoniae * and *Mycoplasma genitalium * in *E. coli *[19], but GFP could not be detected following transformation in *M. gallisepticum *[20]. The chloramphenicol acetyl transferase (CAT) gene has also been used as a selectable marker in *M. pneumoniae * using a modified Tn*4001 * transposon [21].

The *phoA * gene codes for the *E*. *coli * periplasmic alkaline phosphatase (AP), and is active when exported across the cytoplasmic membrane into the periplasmic space [22-24]. Functional alkaline phosphatase is a dimer of two identical subunits and each subunit contains two intramolecular disulfide bridges. The amino-terminal signal sequence is cleaved upon translocation across the cytoplasmic membrane, and the mature PhoA is folded into an active conformation after export to the periplasmic space. Disulfide bond formation is followed by folding into monomers and then conversion to the active dimer conformation [25]. Enzymatic activity of PhoA fusion proteins depends on the presence of an export sequence and this principle has been used in developing reporter vectors to determine membrane protein topology and to facilitate identification of genes involved in bacterial virulence [26].

The aim of this study was to evaluate whether the *E. coli phoA * gene was suitable for use as a reporter gene to investigate gene expression and protein processing in mycoplasmas, using a construct incorporating signal sequences from the *M. gallisepticum * VlhA1.1 lipoprotein and the *ltuf * promoter to express PhoA as a membrane-associated lipoprotein.

## Results

### Construction of plasmid *ltuf *acy *phoA * (pTAP)

The elongation factor Tu promoter region of 277 bp (*ltuf*) (GenBank accession: X16462) and the leader sequence of the *vlh *A1.1 gene (GenBank accession: U90714) from *M. gallisepticum * were originally amplified by PCR from the genomic DNA of *M. gallisepticum * strain S6 and ligated into the pISM2062.2*lac*[14] vector to produce the *ltuf *sig *lac * construct [20]. The *ltuf * promoter region was amplified from *M. gallisepticum * genomic DNA by PCR using the LNF and TSR oligonucleotide primers (Table 1), and the *vlh *A export signal sequence of 51 bp was amplified from *M. gallisepticum * genomic DNA using the TSF and LBR primers (Table 1). These two products were then joined by overlap extension PCR using the primers LNF and LBR. The resultant PCR product was ligated into pGEM-T (Promega) following the manufacturer’s instructions. The *ltuf * promoter and *vlh *A export signal sequence (*ltuf *sig) region was excised from pGEM-T by digestion with *Not *I and * Bam*HI and ligated into similarly digested pISM2062.2*lac* to generate pISM2062.2*ltuf *sig*lac*. Digestion of pISM2062.2*lac * with *Not *I and *Bam *HI resulted in the loss of one inverted repeat region (IR) in the insertion sequence of the transposon.

**Table 1:** Oligonucleotides used in this study

**Oligonucleotide**	**Sequence (5’- 3’)**
LNF	gcggccgcTTTAGGGGTGTAGTTCAATGG
TSR	GTTTTTTCTCTTCATTTTTTTAAATATTTC
TSF	GAAATATTTAAAAAAATGAAGAGAAAAAAC
LBR	ggatccCCAAACGAACCAATACC
LTNF	gccgcggccGCTTTAGGGGTGTAGTTCAATG
SBR	TGTAGTACAACTAGCTGCAGCTAACATTACAAAgGAtCCAATACCTAAT
AXPF	TTAGCTGCAGCTAGTTGTACTACACCTGTTCTAGAAAACCGGGCT
PBgR	CCGaGAT**cta**AAAGGACTG**tta**TATGGCCTTTTTATTTTATTTCAGCCCCAGA
LTPR	CGGTTTTCTAGAACAGGCATTTTTTTAAATATTTC
LTPF	GAAATATTTAAAAAAATGCCTGTTCTAGAAAAC
PBaR	CTTTTTggatc**cta**TTATTTCAGCCCCAGAGC
IRF	GGCCGgGATCAAGTCCGTATTATTGTGTAAAAGTgCtaGc
IRR	ggCCgCtaGcACTTTTACACAATAATACGGACTTGATCcC
GmF	CCAAGAGCAATAAGGGCATAC
GmR	ACACTATCATAACCACTACCG
PRTF	ACGAAAAAGATCACCCAACG
PRTR	GATCCTTTTCCGCCTTTTTC
HLF	TGGTAAGTTAAACGGGATCG
HMR	AATGAACCAGTGATTGTTGGA
UBR	GCAGTAATATCGCCCTGAGC

Lower case indicates changes made to introduce restriction endonuclease cleavage sites and bold lettering indicates the stop codons.

**Figure 1:**
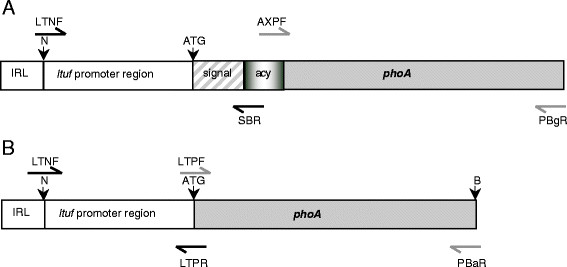
**Schematic representation of * phoA * constructs.A**. The different sections, including the *ltuf * promoter, the signal sequence, the acylation sequence and the *phoA * gene, were combined by overlap extension PCR as described in the text and ligated between the *Not *I and *Bam *HI sites in pISM2062.2. The site of the IR oligonucleotide linker is shown. The positions of the oligonucleotide primers (Table 1) are also shown. **B**. The construct produced containing only the *ltuf * promoter and *pho *A gene. IRL: inverted repeat oligonucleotide linker, N: *Not *I cleavage site, B: *Bam *HI cleavage site, ATG: translational start codon.

The *ltuf * promoter and the *vlh *A1.1 signal sequence from pISM2062.2*ltuf*sig *lac* were amplified by PCR and used to create the *ltuf *acy*phoA * construct. The *ltuf * promoter, *vlh *A1.1 signal and acylation sequence were amplified from pISM2062.2*ltuf *sig*lac * as a single 369 bp product using the primers LTNF and SBR (Table 1). The *Not *I cleavage site was included in the LTNF primer and the *vlh *A signal sequence for lipoprotein export and acylation was included in the SBR primer. The *phoA * gene (1335 bp) was amplified from the plasmid pVM01::Tn* phoA*[27] using the primers AXPF and PBgR (Table 1). Tn*phoA * encodes alkaline phosphatase without the export signal sequence and first five amino acids of the mature protein [24,28]. The 369 bp and 1335 bp PCR products were joined using overlap extension PCR to produce a 1693 bp product using the LTNF and PBgR primers (Figure 1A). The 1693 bp fragment was purified from a 1% agarose gel after electrophoresis using the Qiaex gel extraction kit (Qiagen) and ligated into pGEM-T following the manufacturer’s instructions. An *E. coli * transformant containing a plasmid of the expected size was selected and the insert DNA sequence confirmed using BigDye terminator v3.1 cycle sequencing (Perkin Elmer Applied Biosystems) and the M13 universal primer sites of the vector. The DNA insert was released from the pGEM-T vector by digestion with *Not *I and *Bgl *II, gel purified using the Qiaex gel extraction kit (Qiagen) and ligated into *Not *I and *Bam *HI digested pISM2062.2*lac*[14], resulting in pISM2062.2*ltufacypho *A.

The first 26 bp of the IS256 element, the IR region that was deleted during *Not *I-*Bam *HI digestion of the pISM2062.2 vector, was restored by inserting a 40 bp double stranded linker oligonucleotide, produced by annealing IRF and IRR, into the *Not *I cleavage site of the construct. The linker IRF oligonucleotide contained a mutation at the sixth base (C to G) from the 5' end to inactivate the *Not *I cleavage site, and included an *Nhe *I cleavage site at the 3' end. IRF and the complementary IRR oligonucleotide were annealed by mixing them at equimolar ratios and heating to 50°C for 1 min, then slowly cooling at 1°C/min to 10°C. The double stranded linker had 4 base 5' overhangs at each end to facilitate ligation to *Not *I digested pISM2062.2*ltufacypho *A, resulting in *Nhe *I and *Not *I cleavage sites, and yielding the pISM2062.2*ltufacypho *A vector (pTAP) with the modified IR region.

### Construction of plasmid *ltufphoA * (pTP)

The pISM2062.2*ltufpho *A vector (pTP), which did not contain either the *vlh *A1.1 signal sequence or the acylation sequence of the pTAP plasmid, was also generated. The LTNF and LTPR primers were used to amplify the 305 bp *ltuf * promoter region, whilst the *phoA * gene was amplified using primers LTPF and PBaR. The PCR products were purified and joined by overlap extension PCR using primers LTNF and PBaR, which included *Not *I and *Bam *HI sites, respectively (Figure 1B). The resultant PCR product of 1640 bp was gel purified, ligated into pGEM-T and the DNA sequence confirmed as described above. The *ltufphoA * was released from pGEM-T and ligated to similarly digested pISM2062.2*lac *, resulting in the plasmid pTP, and the IR oligo adaptor then inserted into the *Not *I cleavage site as described above.

### Transformation of *M. gallisepticum * with alkaline phosphatase expression constructs and detection of transformants

Both pTP and pTAP plasmids were used to transform *M. gallisepticum * cells by electroporation. Transformant colonies were observed on MA plates containing gentamicin within 4 days, and colonies picked and grown in MB with gentamicin added. The presence of the gentamicin gene was confirmed by the amplification of a 223 bp PCR product using the oligonucleotide primers GmF and GmR. The genomic location of the transposon in each of the mycoplasma transformants was predicted following genomic DNA sequencing and BLAST searching the *M. gallisepticum * R_low_ genome (Table 2).

**Table 2:** **Site of integration of transposon in *M. gallisepticum * transformants**

**Transformant**	**Gene insertion site**	**Gene annotation**
TAP 3	MGA_0584	Hypothetical protein
TAP 4	MGA_0519	Csn1 family CRISPR-associated protein
TAP 9	MGA_0906	Subtilisin-like serine protease
TP 1	MGA_0552	Hypothetical protein
TP 4	MGA_0554	Hypothetical protein
TP 6	MGA_0816-0817	Hypothetical protein

The *M. gallisepticum- *pTAP transformant colonies on MA plates stained blue following addition of the substrate BCIP/NBT. A strong blue colour development in 10 min was found to indicate transformant colonies, whilst a light blue colour was observed in untransformed colonies only after prolonged incubation. The level of differential staining readily identified pTAP-transformed mycoplasma colonies and those colonies that were larger in size and stained a darker blue colour were selected for subculture and further studies.

### Quantitative RT-PCR

The levels of *phoA * mRNA in both pTP and pTAP transformants were normalised to GAPDH gene expression and the relative abundance determined in three transformants produced using each construct. The difference in gene expression relative to GAPDH mRNA in each transformant was determined. The average level of transcription of *phoA * in each pTAP and pTP transformant was compared. The levels of *phoA * mRNA (mean ± SEM) were determined in pTAP3 (12.49 ± 1.45), pTAP4 (10.89 ± 1.37), pTAP9 (13.41 ± 1.48), pTP1 (1.27 ± 0.05), pTP4 (1.51 ± 0.17) and pTP6 (1.88 ± 0.06). The mean level of *phoA * transcription in pTAP transformants (12.09 ± 0.74) was significantly greater (*P * < 0.05, student’s *t *-test) than in pTP transformants (1.55 ± 0.17).

### Detection and quantitation of alkaline phosphatase activity in pTAP and pTP transformants

Five randomly selected pTAP and pTP transformants were selected and their level of alkaline phosphatase expression determined. The level of AP activity in untransformed cells was used as a baseline. The mean level (± SEM) of AP activity for 5 pTAP transformants was 190 ± 8 U/mg total cell protein, whilst no AP activity was detected in pTP transformants and untransformed cells.

### Alkaline phosphatase expression localized to the plasma membrane

Whole cell proteins from pTAP and pTP transformants were subjected to Western blotting and immunostained using a MAb to alkaline phosphatase. Only in those *M. gallisepticum * transformed with pTAP, and not in those transformed with pTP, was an immunoreactive 47 kDa band observed, indicating PhoA expression. The protein expression of different pTP or pTAP transformants was similar, and the AP expression of representative transformants TAP3 and TP1 are shown in the results.

**Figure 2:**
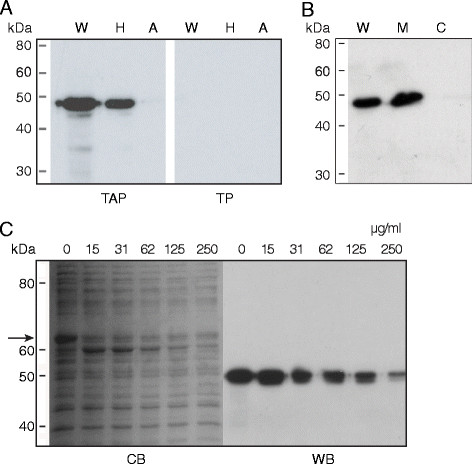
**Immuno-detection of PhoA in fractionated or trypsin treated cellular proteins.A**. Triton X-114 partitioning of *M. gallisepticum * cell proteins. Proteins of pTAP or pTP transformed cells were separated into hydrophobic and aqueous fractions by Triton X-114 partitioning, Western transferred and probed with a MAb to alkaline phosphatase. Panel TAP, *M. gallisepticum * transformed with pTAP and expressing PhoA. Panel **A**, *M. gallisepticum * transformed with pTP cells. Lanes W, whole-cells; H, hydrophobic fraction; A, aqueous fraction. **B**. Immunostaining of cytosolic and membrane fractions of mycoplasma transformants expressing alkaline phosphatase. The fractions were separated on 10 % SDS-polyacrylamide gels, Western transferred and immunostained using a MAb to alkaline phosphatase. Lanes W, whole cells; M, membrane fraction and C, cytosolic fraction. **C**. Surface proteolysis of PhoA. Whole pTAP transformant cells were treated with increasing concentrations of trypsin, the proteins then separated on 10 % SDS-polyacrylamide gels, Western transferred and immunostained using a MAb to AP. Trypsin concentrations (μg/ml) are indicated above each lane. Panels CB, Coomassie brilliant blue stained; WB, Western blot probed with MAb to AP. The arrow indicates the 67 kDa VlhA, which was degraded by increasing concentrations of trypsin. The tryptic products of VlhA can also be seen. Most cellular proteins were minimally affected.

Whole cell proteins of untransformed, pTP-transformed or pTAP-transformed *M. gallisepticum * were subjected to Triton X-114 fractionation and proteins in the hydrophobic and aqueous fractions were separated by SDS-PAGE, transferred to PVDF membranes and immunostained using a MAb to alkaline phosphatase. A band of 47 kDa, corresponding to the predicted molecular weight of expressed PhoA, was observed in the pTAP-transformed whole cell proteins (Figure 2A, TAP, W) and the hydrophobic fraction (Figure 2A, TAP, H), but not in the aqueous fraction (Figure 2A, TAP, A). No reactivity was observed in any of the fractions from pTP-transformed (Figure 2A, TP, W, H, A) or untransformed *M. gallisepticum * cells.

Proteins from *M. gallisepticum * transformed with pTAP were separated into membrane and cytosolic fractions by differential ultracentrifugation and the fractions subjected to SDS-PAGE and Western blotted. Immunostaining with a MAb to alkaline phosphatase detected reactivity in both whole cells (Figure 2B, W) and the membrane fraction (Figure 2B, M), but not in the cytosolic fraction (Figure 2B, C). As a control, MAb 86 [29], against the VlhA membrane lipoprotein, was also used to probe the blot and detected VlhA in both whole cell proteins and in the membrane fraction, but not in the cytosolic fraction (results not shown).

### Trypsin digestion of surface exposed alkaline phosphatase

The cell surface exposure of *M. gallisepticum * proteins and AP were examined by trypsin proteolysis. On the Coomassie blue stained SDS-PAGE gel, the concentration of the major cell surface lipoprotein VlhA decreased with increasing concentrations of trypsin and tryptic products of this lipoprotein could be seen (Figure 2C, CB). Immunostaining of trypsin-treated cell proteins with a MAb to alkaline phosphatase demonstrated a gradual loss of reactivity with increasing concentrations of trypsin from 31 μg/ml to 250 μg/ml (Figure 2C, WB), indicating surface exposure of PhoA. As a control, MAb 86 to VlhA was used to confirm tryptic proteolysis of the surface-exposed VlhA lipoprotein (results not shown). The majority of the proteins detectable by Coomassie blue staining were not affected by trypsin treatment, indicating that cytoplasmic proteins were not exposed to proteolysis.

### Globomycin inhibited PhoA processing

**Figure 3:**
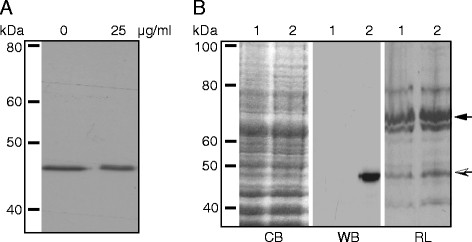
**Lipoprotein processing of PhoA.A**. Effect of globomycin on the processing of PhoA. Mycoplasma transformants were grown in broth without or with globomycin added, as indicated above each lane, and their proteins separated on 10 % SDS-polyacrylamide gels, Western transferred and immunostained using a MAb to AP. In cells grown in globomycin (25 μg/ml), and thus in which signal peptidase II was inhibited, a higher molecular weight band was seen, indicative of the presence of the prolipoprotein. In the absence of globomycin (0 μg/ml) the fully processed 47 kDa lipoprotein is seen. **B**. Radiolabelling of PhoA. *M. gallisepticum * cell proteins and pTAP transformed *M. gallisepticum * cells were radiolabelled with [^14^ C]palmitate and separated on 10 % SDS-polyacrylamide gels. The polyacrylamide gels were stained with Coomassie brilliant blue and autoradiographed or Western transferred and immunostained using a MAb to AP. Lanes 1, *M. gallisepticum * cells; 2, pTAP transformed cells. Panels CB, Coomassie brilliant blue stained; WB, Western transferred and immunostained; RL, radiolabelled and autoradiographed. The dark arrow indicates the 67 kDa VlhA protein and the open arrow indicates the 47 kDa protein.

When pTAP transformant cells were grown with increasing concentrations of globomycin, cell growth was inhibited. A concentration of 25 μg globomycin/ml was the highest to still allow growth of cells. Growth in 25 μg globomycin/ml resulted in an increase in the molecular weight of PhoA (Figure 3A, lane 25 μg/ml) compared to that seen in cells grown in the absence of globomycin (Figure 3A, lane 0 μg/ml).

### Radiolabelling of lipid modified proteins

Lipoproteins of *M. gallisepticum * transformed with pTAP were radiolabelled with [^14^ C]palmitate, separated by SDS-PAGE gel and either stained with Coomassie brilliant blue (Figure 3B, CB) and autoradiographed (Figure 3B, RL) or Western transferred and immunostained (Figure 3B, WB). Following autoradiography, a band of 47 kDa, similar to the expected size of alkaline phosphatase, was detected in the pTAP transformed cells (Figure 3B, RL, 2), suggesting that PhoA in pTAP transformed *M. gallisepticum * was a lipoprotein. A Western blot immunostained with a MAb to AP demonstrated the presence of a recombinant AP protein of similar size to that of the radiolabelled band in pTAP-transformed *M. gallisepticum * (Figure 3B, WB, 2).

### Two-dimensional gel electrophoresis and mass spectrometric analysis of PhoA proteins

**Figure 4:**
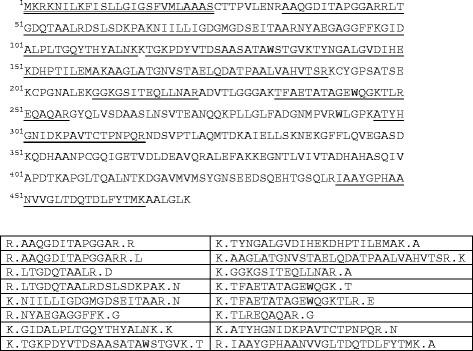
**Identification of PhoA by mass spectrometry.** A tryptic digest of the 2-D gel spot was analysed by MALDI-TOF to obtain a ‘peptide mass fingerprint’ that was subsequently searched against the NCBI database (Taxonomy = Bacteria). The only significant matches were to AP sequences. The sequence shown is PhoA, and the matched peptides are underlined. The predicted signal peptide is double underlined. The 16 matched peptides are shown in the table below.

Following separation of Triton X-114 preparations of protein by 2-D gel electrophoresis, a spot corresponding to PhoA was excised, digested with trypsin and analysed by mass spectrometry. Peptide mass fingerprinting resulted in the positive identification of PhoA, with a Mascot score of 191 and sequence coverage of 53% (Figure 4). Potential (unmodified) amino-terminal tryptic peptides (MKRKNILKFISLLGIGSFVMLAAASCTTPVLENR, CTTPVLENR or SCTTPVLENR) were not identified. Attempts to recover the acylated peptide in organic extracts of the gel spot were also unsuccessful.

## Discussion

In this study we used the transposon Tn*4001 *-based vector pISM2062.2*lac *, modified to form pISM2062.2*ltuf *acy *phoA *, to transform *M. gallisepticum * and express functional alkaline phosphatase on the cell surface. Two constructs containing the alkaline phosphatase gene, one with the *vlhA *1.1 leader and acylation sequences and another without these sequences, were introduced into the Tn* 4001* transposon arm. Following transformation and immunoblotting, a 47 kDa protein was detected in constructs containing the *vlhA *1.1 leader and acylation sequence. The *vlhA * acylation sequence was chosen with the purpose of expressing the recombinant protein as a lipoprotein.

To confirm the processing of PhoA as a lipoprotein, radiolabelling and globomycin treatment of mycoplasma cells were carried out. In *M. gallisepticum *, lipoproteins are predicted to be processed by signal peptidase II, as no other protein processing pathways are known to be present. Processing of lipoproteins by signal peptidase II is specifically inhibited by globomycin and, consequently, processing into a mature lipopeptide is reduced. The increased size of PhoA in cells grown in the presence of globomycin suggests that the VlhA signal sequence was not processed, resulting in an unacylated preprotein. Metabolic labelling of mycoplasmas can be problematic because of the requirement for serum in media, which results in low incorporation of lipids in radiolabelled cells [30]. The presence of other lipoproteins of similar molecular weight that can be labelled with palmitic acid [31] can interfere with specific detection of radiolabelled proteins in SDS-PAGE gels. While it potentially offers greater specificity, detection in 2-D gels was problematic because of the low efficiency of label incorporation, the low abundance of PhoA and the limited loading capacity of 2-D gels, which are likely to have contributed to our inability to detect radiolabelled PhoA after 2-D gel electrophoresis.

Alkaline phosphatase activity was not detected in TP transformants. AP of *E. coli * has two identical subunits, which fold as monomers and then form dimers for enzymatic activity. In *E. coli *, the proteins thioredoxin reductase (TrxB) and thio-disulfide isomerase (Dsb) maintain the reduced state during export of the protein across the cytoplasmic membrane [32]. It is translocated across the membrane via a Sec-dependent pathway to the periplasmic side of the cytoplasmic membrane, the leader peptide is cleaved and the mature alkaline phosphatase is released into the periplasm [33]. Homologous proteins must have been present to enable the folding and export of functional PhoA in pTAP-transformed *M. gallisepticum *. The absence of detectable alkaline phosphatase expression and activity in pTP-transformed mycoplasma cells could be attributable to the lower level of transcription of *phoA * together with the possible retention of the protein in the cytoplasm in a reduced form, and thus inactive, and subsequent proteolysis. Since the promoter region and all other sequences preceding the start codon were identical to those in pTAP, similar levels of transcription were expected for both constructs, but there was an eight-fold lower level of *phoA * in pTP transformed cells compared to in those transformed with pTAP. It is not clear whether the signal sequence in the pTAP construct could have affected transcription and further studies are needed to elucidate the mechanisms for the lack of PhoA activity in pTP transformants.

Generally the differences in the protein export pathway of Gram-positive bacteria result in low *phoA * activity when it is introduced into these organisms [34]*.* This has led to the use of the *Enterococcus faecalis *-derived *phoZ * as a reporter system in Gram-positive bacteria [35]. Although mycoplasmas have similarities to Gram-positive bacteria, this study has shown that *phoA * from *E. coli * can be expressed as a membrane protein in *M. gallisepticum *.

As the construct could be successfully introduced into *M. galliseptcium * using the transposon Tn* 4001*, it could provide a suitable model for investigating membrane protein export in other mycoplasma species. Other workers have investigated the use of Tn* phoA* to detect membrane protein export signal sequences from genomic libraries of mycoplasmas, after introduction into *E. coli*[13,36]. The pTAP vector will be a valuable and versatile tool for studies analysing regulatory effects of promoter regions, gene expression using different translational start codons and leader sequences and also for optimising expression of foreign antigens. Studies on gene regulation could also be facilitated by using the PhoA vector.

Mycoplasma lipoproteins are surface exposed and have atypical acylation, and are commonly immunodominant. Thus expression of an antigen as a lipoprotein is likely to be an optimal approach to inducing a vaccinal response [37]. Heterologous lipoprotein expression has been demonstrated in mycoplasmas and its use as live vaccine was emphasized in *Mycoplasma capricolum * subsp. *capricolum *, in which spiralin has been expressed on the cell surface using an *oriC * plasmid vector [38]. The *phoA * vector we have described here could be used to facilitate optimisation of expression of heterologous bacterial or viral antigens, and immunomodulators on the mycoplasma cell surface. Preliminary studies in our laboratory using the *phoA * vector have been successful in expressing the immunomodulatory genes of chicken IFN-γ in the ts-11 vaccine strain [39]. The expression of such immunomodulatory genes has the potential to enhance the immunogenicity of live attenuated vaccines by intrinsic adjuvantation. The *phoA * expression system allows rapid assessment of the level of expression from different promoter and signal sequences and thus optimisation of both expression and translocation of such heterologous proteins.

## Conclusions

This is the first study to express alkaline phosphatase on the mycoplasma cell surface. The use of this system will enable us to further study protein translocation across mycoplasma membranes. The study also demonstrates the ease of using *phoA * as a reporter gene in mycoplasmas. Thus, we have successfully developed a vector system in mycoplasmas with the potential for use in optimising heterologous gene expression and ultimately in recombinant vaccine development, in addition to its potential as used as a tool in studies of the molecular pathogenesis of mycoplasmosis.

## Methods

### Bacterial strains and culture conditions

*M. gallisepticum * strain S6 was grown in mycoplasma broth (MB) or on mycoplasma agar (MA; containing 1% agar (Oxoid) without phenol red) at 37°C [29]. For selection of mycoplasma transformants, 16 μg of gentamicin/ml (Invitrogen) was added to the media.

*E. coli * DH5α cells were used as the host for genetic manipulation and cloning of plasmids. Clones were grown in Luria-Bertani broth (LB) or on LB agar plates (LB with 1% agar) containing 100 μg ampicillin/ml (Amresco) at 37°C. For detection of alkaline phosphatase activity in transformants grown on solid media, the substrate 5-bromo-4-chloro-3-indolyl phosphate (BCIP) (Sigma) was added to the LB agar plates or MA to a final concentration of 40 μg/ml.

### Amplification of DNA sequences by PCR

PCR was carried out using Platinum HiFi *Taq * DNA polymerase (Invitrogen) in a 25 μl volume containing 2.5 μl of 10 x buffer (Invitrogen), 2 mM MgSO_4_, 100 μM of each deoxynucleotide triphosphate (Bioline), 0.4 μM of each primer, 1.5 U of enzyme and 5 ng of each PCR product as template. The reaction was performed in an iCycler (BioRad) with an initial cycle of 95°C for 3 min, followed by 35 cycles of 94°C for 30 s, 60°C for 30 s and 72°C for 1 min/kb, with a final extension at 72°C for 7 min.

### Development of alkaline phosphatase construct

The *E. coli phoA * gene lacking a promoter, signal sequence and the first 5 residues of the mature protein [28] was cloned under the control of the *ltuf * promoter and fused to the lipoprotein acylation signal sequence of *vlh *A1.1, and subsequently cloned into the Tn*4001 * transposon contained in pISM2062.2 to generate the plasmid, pISM2062.2*ltuf *acy*phoA * (pTAP) (Figure 1A). Another plasmid (pTP), lacking the signal and acylation sequence (pTP), was also produced (Figure 1B).

The pTAP and pTP constructs were introduced into *E. coli * DH5α by electroporation using a Gene Pulser (BioRad) with settings of 2.5 kV and 25 μF. Recombinants were selected for ampicillin resistance and clones were screened for the presence of the gentamicin resistance gene using the oligonucleotide primers GmF and GmR. Selected clones were cultured in larger volumes and plasmid DNA extracted using a Midi prep kit (Qiagen) according to the manufacturer’s instructions.

### Transformation of * M. gallisepticum*

*M. gallisepticum * was transformed by electroporation as described previously [39,40]. Following electroporation, cells were gently resuspended in 1 ml of ice-cold MB, incubated at 37°C to allow expression of the gentamicin resistance gene, then a 500 μl aliquot of the culture inoculated onto MA plates containing 16 μg of gentamicin/ml, which were allowed to dry and then incubated at 37°C for 4 days. The plates were examined for colony development and single colonies selected and subcultured in MB containing 16 μg of gentamicin/ml.

### Detection of alkaline phosphatase activity on MA plates

To detect alkaline phosphatase activity in colonies of transformed *M. gallisepticum * on MA plates, a single tablet of BCIP/nitroblue tetrazolium (NBT) (Sigma Fast, Sigma) was dissolved in 3 ml distilled water and sprayed onto the colonies uniformly as a thin layer using a pump atomizer. After 10 min colonies were observed for the presence of a blue colour.

### Genomic DNA sequencing

To determine the insertion site of the transposon, genomic DNA sequencing was carried out using the ABI Prism BigDye Terminator v3.1 (BDT) sequencing system (Perkin Elmer Applied Biosystems) and the UBR oligonucleotide primer (Table 1) according to the manufacturer's instructions, with minor modifications. Approximately 2 μg of genomic DNA was combined with 1 μM of the UBR oligonucleotide, 4 μl of the BDT enzyme mixture, 4 μl of 5 x BDT buffer and distilled water to 20 μl. The sequencing reaction mixture was incubated at 96°C for 5 min, then through 60 cycles of 96;°C for 30 s, 50°C for 10 s and 60°C for 4 min in an iCycler thermocycler (BioRad). The sequencing products were purified according to the manufacturer’s instructions using ethanol-EDTA-sodium acetate precipitation and analysed using an ABI3100 capillary sequencer.

### Quantitative RT-PCR

Quantitative RT-PCR (qRT-PCR) was used to determine the level of transcription of the *phoA * gene in each of the transformants. To achieve this, total RNA from 6 ml of transformant cells was extracted using an RNA purification kit (Qiagen), following the manufacturer’s instructions. The total amount of RNA was determined using an ND-1000 spectrophotometer (NanoDrop). To remove any contaminating DNA, 2 μg of extracted RNA was treated with 2 U of DNase I (Invitrogen) in a buffer containing 2 μl of 10 x DNase I buffer and RNase-free water in a total volume of 20 μl for 15 min at room temperature. To produce cDNA, 1 μg of DNAse I-treated RNA was used. For each 1 μg of DNAse I-treated RNA, 50 ng of random hexamers (Invitrogen) and 10 nM of each deoxynucleoside triphosphate (dNTPs, Bioline) were added and the mixture incubated at 65°C for 5 min, then immediately cooled on ice. To this, 4 μl of 5 x first strand reaction buffer (Invitrogen) and dithiothreitol (Invitrogen) to a final concentration of 0.1 M were added and the mixture incubated at 25°C for 2 min, then 1 μl (200 U) of Superscript II reverse transcriptase (RT) (Invitrogen) was added and the reaction incubated for 10 min. A negative control (no RT) was also included, with 1 μl of RNase-free water substituted for the Superscript II reverse transcriptase. The reverse transcription reactions were incubated at 42°C for 50 min. The reaction was stopped by incubation at 70°C for 15 min and the total volume made up to 600 μl with nuclease-free water and aliquots stored at −20°C. Each qRT-PCR reaction was conducted in a 20 μl volume and contained 5 μl template cDNA, 10 μl of 2 x Platinum SYBR Green qPCR Supermix containing Rox Dye (Invitrogen) and 100 nM each of the PRTF and PRTR primers (Table 1). Reactions were run using a Stratagene MX3000P. Each assay included test cDNA, the no-RT control reaction previously described and a no template control, to which only water was added. The cycling conditions were an initial incubation for 2 min at 50°C, followed by 5 min at 95°C, then 40 cycles of 95°C for 30 s and 60°C for 30 s. Reactions were carried out in triplicate for each sample. Relative quantification of *phoA * transcription was normalised against transcription from the glyceraldehyde 3-phosphate dehydrogenase gene (GAPDH, GeneID: 1090024) using the HLF and HMR primers (Table 1) and the relative level of expression calculated using the delta-delta Ct method [41].

### Detection of alkaline phosphatase activity in cultured cells

Mycoplasma transformants were grown in 10 ml MB supplemented with gentamicin at 16 μg/ml until an approximate pH of 7.2 was reached, then pelleted by centrifugation at 20,000 x g for 20 min at 4°C. The cells were resuspended and washed twice in ice-cold 0.05 M Tris, pH 8.0 (T buffer) and again centrifuged and washed as before. The cells were finally resuspended in T buffer with 1% Triton X-100 (ICN) added and incubated for 15 min at 4°C. The total protein concentration of the cell lysate was determined in triplicate using a BCA kit (Pierce) following the manufacturer’s instructions. To determine the AP activity of each transformant in triplicate, 10 μl of the cell lysate was added to reaction buffer (1 M Tris, pH 8.0, 1 mM MgCl_2_) to which 50 μl of 2 mM disodium p-nitrophenyl phosphate (pNPP, Calbiochem) in reaction buffer was added and the mixture incubated at 37°C for 30 min. The reaction was terminated by addition of 100 μl 2 M NaOH and the absorbance read at 410 nm using a spectrophotometer (Labsystems Multiskan MS). In each assay doubling dilutions in triplicate, starting at 12 U of bacterial alkaline phosphatase (BAP, Invitrogen), were made in T buffer and similarly treated, enabling a standard curve to be constructed. The alkaline phosphatase activity in the BAP dilution series was plotted against absorbance and this used to determine the alkaline phosphatase activity in each sample, which was expressed as BAP U/mg of total cell protein.

### SDS-PAGE, Western blotting and immunostaining

Mycoplasma cell proteins were separated by SDS-PAGE as described previously [42]. The protein concentrations of mycoplasma cells were determined using the Pierce BCA protein assay kit (Thermo Scientific), using bovine serum albumin as the standard, and 10 μg of total cell protein was loaded into each well of a polyacrylamide gel. After separation in a 10% polyacrylamide gel, proteins were transferred onto PVDF membranes and incubated in blocking solution containing 5% (w/v) skim milk (Devondale) in PBS with 0.1% (v/v) Tween 20 (PBS-T) for 2 h at room temperature on a rocking platform. Following blocking, membranes were washed three times for 5 min each in PBS-T. Membranes were then incubated for 1 h with mouse monoclonal antibody (MAb) to alkaline phosphatase (Chemicon) at a 1:5000 dilution in blocking solution. The membranes were washed thrice for 5 min with PBS-T and incubated with rabbit anti-mouse-horseradish peroxidase (HRPO) conjugate (Dako) for 1 h at a 1:5000 dilution in blocking solution. This was followed by washing thrice for 5 min each with PBS-T and bound conjugate was then detected by chemiluminiscence using an ECL Plus kit (GE Healthcare) according to the manufacturer's recommendations. As molecular weight marker, 10 μl of biotinylated protein ladder (Cell Signaling Technology) was loaded, and for detection in Western blots, HRP-linked anti-biotin antibody was used.

### Partitioning of mycoplasma cell proteins into hydrophobic and aqueous fractions using Triton X-114

Mycoplasma cell proteins from a 20 ml overnight culture were separated into hydrophobic and aqueous fractions using the detergent Triton X-114 (Sigma) [43,44]. The urea solubilised protein fractions were then analysed by SDS-PAGE.

### Membrane and cytoplasmic separation

Membrane and cytoplasmic fractions of *M. gallisepticum * were purified essentially as previously described for *M. pneumoniae *[45]. The cytosolic and membrane fractions were then analysed by SDS-PAGE and immunoblotting.

### Trypsin treatment of intact *M. gallisepticum * transformant cells

*M. gallisepticum * cells were cultured and the cell pellet washed in 50 mM Tris, 0.145 M NaCl, pH 7.4 (TS buffer). This was repeated twice and the cells finally resuspended in 600 μl TS buffer, then divided into 6 equal aliquots. A dilution series of trypsin (Sigma) at 250, 125, 62, 31 and 15 μg/ml was made in TS buffer and 100 μl of each dilution, as well as a control without any trypsin, added to a separate aliquot of cells and these incubated at 37°C for 30 min. Digestion was stopped by the addition of 200 μl of 0.125% (w/v) trypsin inhibitor (Sigma). The trypsin-treated cells were collected by centrifugation, resuspended in TS buffer and proteins in the sample separated by SDS-PAGE and either stained with Coomassie brilliant blue or immunoblotted.

### Globomycin treatment

Globomycin is a peptide antibiotic that inhibits the processing of prolipoprotein to mature lipoprotein by signal peptidase II [46,47]. Mycoplasma cells were grown in the presence or absence of globomycin (a gift from Dr. M. Inukai, IUHW, Japan), dissolved in methanol. Cells were grown in MB with 25 μg globomycin/ml and the cells were harvested by centrifugation at 20,000 x g for 20 min at 4°C, washed thrice in PBS and proteins in the sample separated by SDS-PAGE and either stained with Coomassie brilliant blue or immunoblotted.

### Radiolabelling of *M. gallisepticum * lipoproteins

*M. gallisepticum * transformants were cultured in 20 ml MB to pH 7.2 and cells harvested and resuspended in 2 ml of fresh MB containing 10 μCi [^14^ C]palmitate/ml (Perkin Elmer), then incubated at 37°C for 18 h. The cells were centrifuged at 8000 *g * for 20 min at 4°C and washed in 2 ml PBS. The washing step was repeated three times. The cells were resuspended in 100 μl PBS and SDS-PAGE lysis buffer added. Mycoplasma proteins, together with [^14^ C] methylated molecular weight markers (Sigma), were separated by SDS-PAGE in a 10% polyacrylamide gel and fixed in a solution of 10% (v/v) glacial acetic acid and 30% (v/v) methanol for 30 min. The gel was incubated in EN^3^HANCE (Life Science Products) according to the manufacturer's instructions, vacuum dried and then exposed to X-ray film (Kodak).

### Two-dimensional gel electrophoresis of fractionated mycoplasma cell proteins

*M. gallisepticum * cells were harvested and fractionated with Triton X-114 as described above, and the hydrophobic fraction was resuspended in 8 M urea, 2% CHAPS, 0.5% IPG buffer (3–10) and 18 mM dithiothreitol (DTT, GE Healthcare). A 125–150 μg sample of protein, as estimated using the 2-D-Quant kit (Amersham Biosciences), was subjected to isoelectric focusing (IEF) on 7 cm strips over the pH range of 3–10 (GE Healthcare) using the following parameters: rehydration at 30 V for 6 h, 60 V for 6 h; running at 200 V for 1 h, 500 V for 1 h, 1000 V for 1 h, 1000–8000 V for 1 h and 8000 V for 1.5 h. After isoelectric focusing the gel strips were equilibrated twice in 6 M urea, 75 mM Tris–HCl, pH 8.8, 2% SDS and 30% glycerol (65 mM DTT, 0.135 M iodoacetamide) for 15 min each. Immediately following equilibration and fixing, the IEF strips were transferred onto a 10% SDS-polyacrylamide gel and fixed in place with 0.5% agarose containing bromophenol blue. Electrophoresis was carried out at 200 V for 1 h. The gels were stained with Coomassie brilliant blue.

### Mass spectrometry of PhoA

Following 2-D gel electrophoresis of fractionated cellular proteins of untransformed and TAP- transformed *M. gallisepticum *, the gel images were compared in order to locate the gel spot likely to correspond to PhoA. This spot was excised and subjected to in-gel digestion and peptide mass fingerprint analysis as described previously [48] using an Ultraflex III MALDI TOF/TOF instrument (Bruker Daltonics, Bremen). Spectra were acquired in reflectron mode and calibrated externally using a standard peptide mix (Bruker Daltonics). Proteins were identified using Mascot v 2.2 (Matrix Science) with the following search parameters: database = NCBI*,* taxonomy = bacteria, enzyme = trypsin, mass tolerance = 30 ppm, missed cleavages = 1, fixed modifications = carbamidomethyl (Cys) and optional modifications = oxidation (Met).

## Competing interests

The authors declare that they have no competing interests.

## Authors’ contributions

I.S.P designed the study, performed the experiments and data analysis, and drafted the manuscript, AK helped with the experiments, CC contributed the *ltuf *sig*lac * construct, P.D.V and M.D.G performed mass spectrometry identification and analysis and provided suggestions about the manuscript. G.F.B and P.F.M contributed to the study design, analysis, drafting and review of the manuscript. All authors have read and approved the manuscript.
